# Assessment of disease activity control and evaluation strategy in patients with Takayasu arteritis undergoing cardiac surgery: a retrospective cohort study

**DOI:** 10.3389/fcvm.2026.1770394

**Published:** 2026-06-03

**Authors:** XinPei Liu, Di Wu, Jun Zheng, ChaoJi Zhang, ShangDong Xu, XingRong Liu, GuoTao Ma, Sheng Yang, JianZhou Liu, Qi Miao

**Affiliations:** 1 Department of Cardiac Surgery, Peking Union Medical College Hospital, Beijing, China; 2 Department of Rheumatology, Peking Union Medical College Hospital, Beijing, China; 3 Department of Surgery, TEDA International Cardiovascular Hospital, Tianjin, China

**Keywords:** cardiac surgery, prognosis, reoperation, rheumatic disease, Takayasu's arteritis

## Abstract

**Introduction:**

For patients with Takayasu’s arteritis (TAK) requiring cardiac surgery, optimizing preoperative disease activity control and assessment is pivotal to improving surgical outcomes; however, an established strategy to minimize activity-driven adverse events remains undefined. Following the 2014 implementation of an institutional, multidisciplinary TAK activity control and evaluation strategy, this study aimed to robustly assess its clinical safety.

**Methods:**

This single-center retrospective cohort study included 64 patients with TAK and cardiac involvement who underwent cardiopulmonary bypass surgery under this multidisciplinary protocol. Propensity score-matched non-TAK (NTAK) patients undergoing identical surgeries during the same period served as controls. Outcomes were short-term mortality/complications and mid-term survival/complications across a mean follow-up period of 4.1 years.

**Results:**

The TAK cohort demonstrated a higher incidence of surgical site complications than controls (17.19% vs. 0%, *P* = 0.003). Early postoperative mortality/complication rates and mid-term survival during follow-up showed no difference. However, patients with TAK had a significantly higher incidence of cardiovascular re-hospitalization [subdistribution hazard ratio (sHR): 3.487; 95% CI: 1.414–8.559; *P* = 0.007] and new cardiovascular surgery (sHR: 6.342; 95% CI: 1.325–30.347; *P* = 0.021). Crucially, the risk of structural reoperation driven by the initial surgical failure did not differ significantly between cohorts (sHR: 1.077; 95% CI: 0.151–7.705; *P* = 0.941).

**Discussion:**

Guided by a systematic, multidisciplinary disease activity control and evaluation strategy, cardiac surgery via cardiopulmonary bypass for patients with TAK was not associated with increased mortality or major perioperative complications compared to matched NTAK controls. Although patients with TAK face elevated long-term risks of recurrent cardiovascular events and future interventions, the risk of initial surgical failure was not augmented relative to matched NTAK controls.

## GRAPHICAL ABSTRACT

**Figure:**
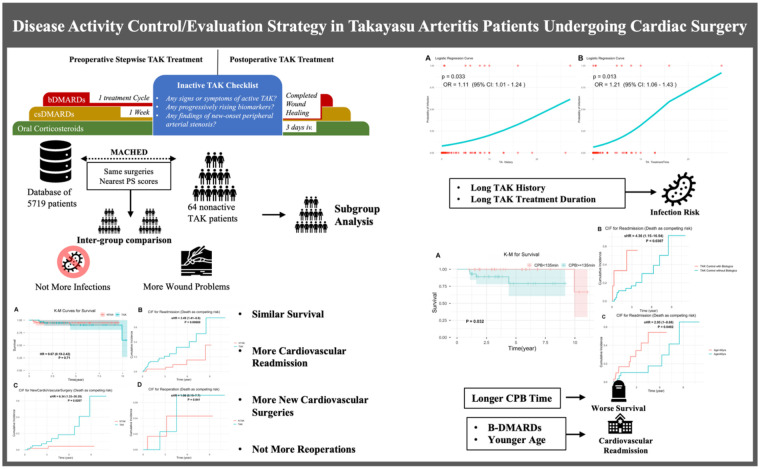

## Introduction

Takayasu's arteritis (TAK) is a large-vessel vasculitis that predominantly occurs in Asia and the Middle East, presenting with a global incidence of 1.11/million person-years and a notable female-to-male (F/M) ratio of 6:1 (1). The pathology primarily affects the aorta and its major branches. Consequently, patients frequently require cardiac surgery to manage severe secondary sequelae—–such as aortic valve regurgitation, aortic aneurysms, or coronary artery involvement—with an estimated surgical intervention rate of 19.7% (2).

Attendant to persistent systemic inflammation and concurrent chronic corticosteroid therapy, patients with TAK are widely recognized to have an augmented risk of perioperative and postoperative complications following major cardiac procedures (3). Although prior investigations have attempted to characterize these clinical risks (4–6), the existing literature remains constrained by critical limitations. Specifically, published TAK cohorts often lack standardized preoperative protocols for systemic disease activity control and assessment. Furthermore, from a methodological standpoint, these foundational studies often have inadequate sample sizes and control-cohort designs.

To address these historic limitations, since 2014, a dedicated multidisciplinary team comprising cardiac surgery, rheumatology, and critical care specialists at our center has systematically implemented a structured, stepwise preoperative pharmacological protocol alongside standardized disease activity assessment criteria for patients with TAK requiring cardiac intervention. The primary objective of the present study was to rigorously evaluate the clinical safety and efficacy of this multidisciplinary strategy by assessing whether its implementation effectively mitigates perioperative and postoperative complication risks in patients with TAK, reducing them to levels comparable with matched non-TAK controls.

## Methods

### Patients

**Figure 1:**
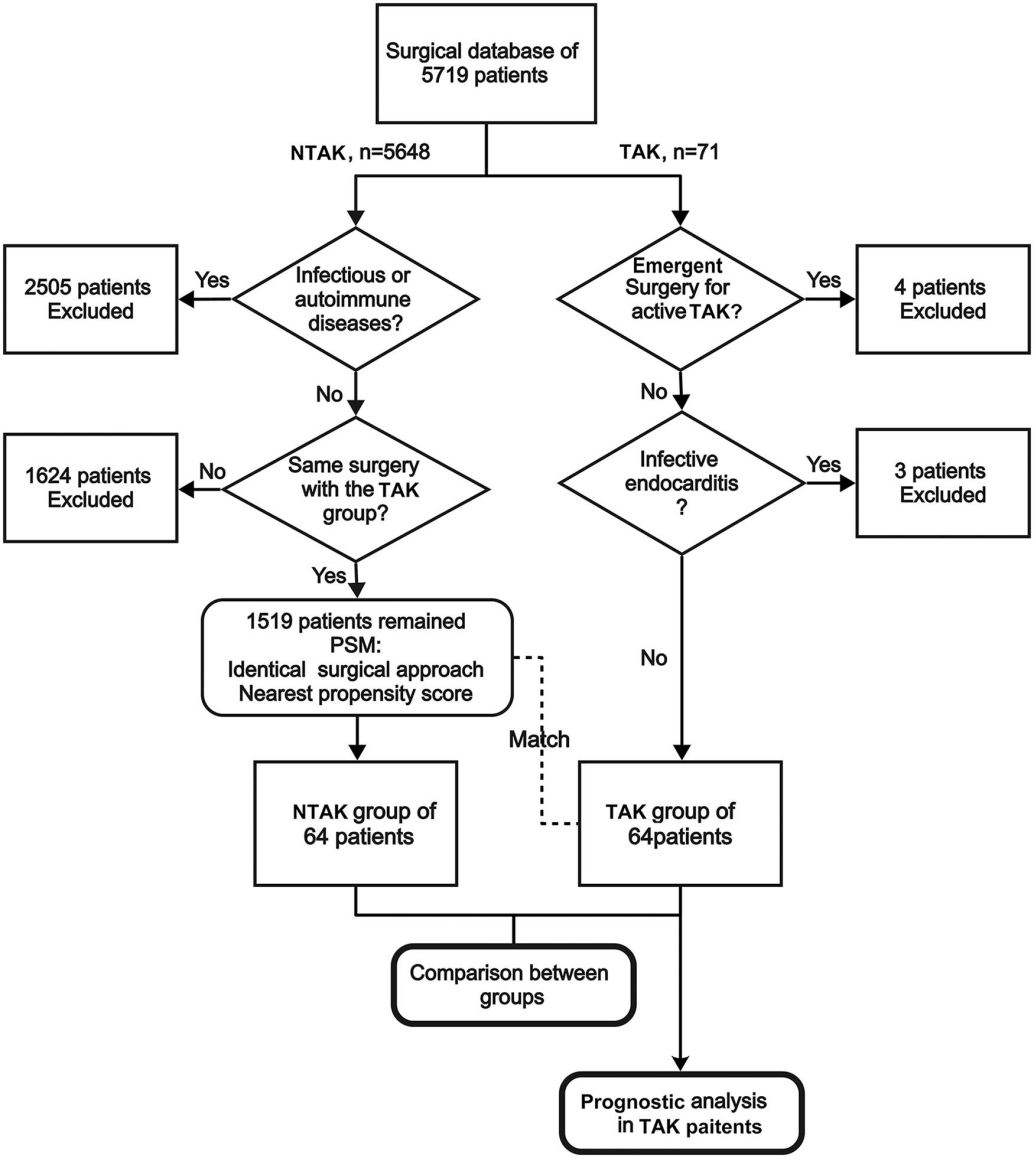
The patient selection process of this study.

This retrospective cohort study was approved by the Institutional Review Board (Approval No. I-22PJ1016). All participants provided written informed consent prior to data collection. To evaluate the clinical outcomes of patients with TAK managed under the multidisciplinary framework relative to non-TAK controls, we screened our database to identify patients with TAK who underwent cardiac surgical intervention between January 2014 and January 2024. Inclusion criteria required patients to fulfill the following conditions: (1) meeting the 1990 American College of Rheumatology (ACR) diagnostic criteria for TAK (7); (2) exhibiting preoperative disease activity status assessed as “inactive” according to our standardized institutional protocol; and (3) having histopathological confirmation of TAK from surgical specimens. Patients were excluded if they: (1) underwent emergent surgery without undergoing the protocol treatment/assessment beforehand, or (2) had a comorbid autoimmune disease or infective endocarditis. Consequently, 64 eligible patients with TAK comprised the definitive study cohort. (Figure 1 illustrates patient selection.)

A matched non-TAK control group was generated from 1:1 propensity score matching (PSM) from the identical institutional database. Potential controls were excluded if they: (1) were presented with active preoperative infectious/autoimmune diseases, or (2) underwent a surgical approach that differed from that of their intended matched TAK case. Subsequently, we calculated propensity scores [PSM variables in (Table 1)]. Each patient with TAK was then individually paired with an NTAK control based on matching the identical surgical procedure codes and the closest linear propensity score using the nearest-neighbor caliper matching method.

**Table 1:** Baseline characteristics before PSM.

Variables	TAK	NTAK	*P* value
Sex (male)	25.0%	76.7%	<0.001^a^
Age	38.16 ± 12.81	59.38 ± 11.43	<0.001^a^
BMI	23.10 ± 4.09	25.21 ± 3.47	<0.001^a^
DM needs insulin	3.1%	35.4%	<0.001^a^
Peripheral vascular stenosis	50.0%	11.4%	<0.001^a^
Af	4.7%	6.6%	0.552
CCr	104.48 ± 36.49	86.11 ± 33.35	<0.001^a^
LVEF^b^	1[1,1]	2[1,2]	<0.001^a^
PASP^b^	1[1,1]	1[1,1]	0.9
NYHA	2[2,2.25]	2[2,2]	0.699
Immobilized	12.5%	20.1%	0.138
COPD	4.7%	5.8%	0.705
Redo	7.8%	1.1%	<0.001^a^

AF, atrial fibrillation; BMI, body mass index; CCr, creatinine clearance; COPD, chronic obstructive pulmonary disease; DM, diabetes mellitus; LVEF, left ventricular ejection fraction; PASP, pulmonary artery systolic pressure.

a The difference is statistically significant.

b In the analysis, LVEF was categorized as an ordinal variable using thresholds of >50%, 31%–50%, 21%–30%, and ≤20%; PASP was transformed into an ordinal variable with gradations of normal, 31–55 mmHg, and >55 mmHg.

### Interventions

**Figure 2:**
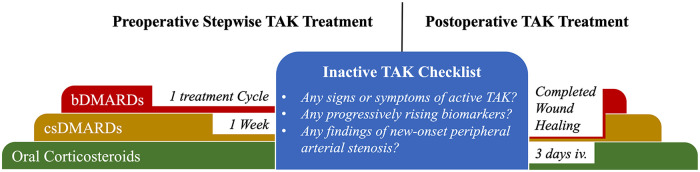
Perioperative management protocol for patients with TAK undergoing cardiac surgery. This schematic illustrates: (1) Stepwise therapy for preoperative disease activity control; (2) clinical criteria for inactive TAK; (3) postoperative intravenous corticosteroids protocol; and (4) the timing of withholding and resumption of medical therapies during the perioperative period.

Perioperative disease control for patients with TAK undergoing cardiac surgery is shown in Figure 2. All patients with TAK received rheumatology follow-up once surgery was indicated. Rheumatologists managed all perioperative TAK disease activity. The preoperative protocol consisted of repeated clinical and biomarker assessments alongside stepwise therapy escalation for inadequate control: initiating with corticosteroids alone, escalating to corticosteroids combined with csDMARDs (including but not limited to methotrexate, cyclophosphamide, leflunomide, cyclosporine, and tacrolimus), and if still inadequate, biologics [bDMARDs, including tumor necrosis factor-α (TNF-α) inhibitors, interleukin-6 (IL-6) receptor antagonist, and CD20-directed cytolytic monoclonal antibodies] (8, 9) were added.

Surgery was only scheduled after rheumatologists assessed the disease as “inactive” in the context of baseline immunosuppression with prednisone monotherapy (<20 mg/day), based on predefined criteria: (1) Absence of new active TAK symptoms (e.g., claudication, acute vision changes, stroke, paresis), (2) stable biomarkers (ESR, CRP, IL-6, TNF-α), and (3) no new stenotic lesions on vascular Doppler ultrasound.

Preoperatively, csDMARDs were discontinued for 1 week; bDMARDs were paused for 1–2 dosing intervals, depending on distinct pharmacodynamic profiles and infection risk stratification. Cardiac surgeries performed are listed/detailed in Supplementary Table S1. During surgery and the first three postoperative days, patients received 100–200 mg of intravenous hydrocortisone daily (10), titrated against the preoperative oral corticosteroid regimen, usually receiving <20 mg/day prednisone, and then resumed preoperative oral corticosteroids. csDMARDs/bDMARDs were reintroduced after complete wound healing.

### Data collection and analysis

The primary predictor was TAK diagnosis according to the 1990 ACR criteria. Additional predictors included TAK history, TAK treatment and treatment duration, peripheral vascular stenosis, preoperative biomarkers (CRP, ESR, IL-6, TNF-α), and pathological evidence of active disease (Supplementary Table S2). TAK treatment was defined as the most intensive regimen administered for TAK before admission, including corticosteroids, conventional synthetic disease-modifying antirheumatic drugs (csDMARDs), and biologic disease-modifying antirheumatic drugs (boDMARDs). TAK treatment duration was defined as the period from the patient's most recent regular TAK treatment before surgery. Peripheral vascular stenosis was defined as severe stenosis identified by ultrasound or CTA. Pathologically active findings in TAK were defined by the presence of granulomatous lesions with transmural inflammation and destruction of the muscle–elastic lamina within the tunica media. Surgical specimens were independently reviewed by two cardiovascular pathologists blinded to the patients’ clinical status; discrepant cases were adjudicated by a third senior pathologist.

The primary outcomes were early and mid-term mortality. Secondary outcomes included postoperative complications, cardiovascular readmission, new cardiovascular surgery, and reoperation. Early death was defined as death occurring during hospitalization or within 30 days post-surgery. Postoperative infection was defined as any infection requiring escalation of antibiotic therapy beyond prophylactic cefuroxime, regardless of bacteriological findings. New cardiovascular surgery referred to surgical intervention for newly occurring major cardiovascular events after the initial procedure, while reoperation referred to repeat surgery due to failure of the initial procedure.

In addition to group comparisons between the TAK and control groups, prognostic factor analyses were performed to identify predictors of outcomes among patients with TAK.

For statistical analyses, LVEF was categorized as an ordinal variable using the following thresholds of >50%, 31%–50%, 21%–30%, and ≤20%. PASP was similarly categorized into three ordinal groups: normal, 31–55 mmHg, and >55 mmHg. Statistical analyses were conducted using (R, Version 4.5.1). Specifically, binary variables were expressed as percentages and compared using the chi-square test or Fisher's exact test. Continuous variables with normally-distributed data were presented as mean ± SD and compared using the independent *t*-test, whereas non-normally distributed variables were expressed as median (IQR) and compared using the Mann–Whitney U test. Post-PSM comparisons were performed using paired *t*-tests for continuous variables or McNemar's test for categorical variables. Survival outcomes were evaluated using Kaplan–Meier curves with the log-rank test. Complication incidence analysis employed cumulative incidence function (CIF) curves, with death treated as a competing risk, and the curves were compared using the Fine–Gray test.

## Results

### Descriptive statistics (TAK group)

The 64 patients with TAK had a mean age of 38.3 ± 13.0 years and were predominantly women (75%). The median EuroSCORE II risk score was 3.04%. The median interval between TAK diagnosis and surgery was 1.75 years (IQR, 0.41–7.00 years), whereas the median duration of preoperative TAK-specific medical therapy was 0.75 years (IQR, 0.17–5.25 years). Regarding preoperative immunomodulatory therapy, 25.0% received corticosteroids alone, 53.1% received csDMARDs, and 10.9% received bDMARDs. The remaining 10.9% were newly diagnosed with TAK upon admission and were assessed as having inactive without prior treatment.

Ultrasound findings of severe peripheral arterial stenosis were prevalent, occurring in 76.6% of patients. Furthermore, although although all patients met the predefined surgical criteria of clinical stability without sustained inflammatory progression, at least one inflammatory biomarker remained elevated preoperatively in 60.9% of patients.

Common intraoperative observations included ascending aortic thickening (75.0%) and tissue adhesions (20.3%). Histopathological examination of surgical specimens demonstrated evidence of active arteritis in 12.5% of patients, despite these patients fulfilling predefined preoperative clinical and biomarker criteria for inactivity preoperatively.

Patients underwent a total of 13 distinct cardiac surgical procedures. Aortic valve replacement (AVR) was the most common procedure (76.6%), often performed concurrently with ascending aorta replacement (35.9%), aortic arch replacement (25%), root replacement (18.8%), or coronary artery bypass grafting (CABG; 18.8%). The mean cardiopulmonary bypass time was 143.3 ± 58.4 min. Concomitant peripheral vascular interventions were performed in eight patients (12.5%).

Early mortality was 3.1% (*n* = 2). The overall incidence of postoperative infection requiring escalation of antibiotic therapy occurred in 14.1% of patients (*n* = 9), whereas wound complications were observed in 17.2% (*n* = 11) of patients with TAK. During a mean follow-up period of 4.1 ± 2.6 years, all-cause mid-term mortality was 8.5%. Cardiovascular readmission occurred in 28.8% of patients (17/59), among whom 82.4% of readmissions (14/17) were driven by non-index-surgery-related events. New cardiovascular surgical procedures were required in 17.0% of patients (10/59), whereas redo surgeries due to failure of the initial operation were required in 3.4% of patients (2/59).

### Propensity score matching

Among the 1,519 non-TAK patients in the database, we successfully identified 64 propensity score-matched controls at a 1:1 ratio. The significant baseline differences observed before matching (Table 1) were effectively mitigated by the matching process, as demonstrated by balanced covariate distributions in Table 2, with all standardized mean differences (SMDs) < 0.1 and non-significant *P*-values, thereby reducing potential confounding.

**Table 2:** Baseline characteristics after PSM.

Variables	TAK	NTAK	*P* value	SMD
Sex (male)	25.0%	28.1%	0.689	0.074
Age	38.16 ± 12.81	38.92 ± 12.56	0.733	0.061
BMI	23.10 ± 4.09	23.33 ± 3.43	0.729	0.067
DM needs insulin	3.1%	1.6%	1	0.033
Peripheral vascular stenosis	50.0%	46.9%	0.724	0.099
Af	4.7%	6.3%	1.000	0.063
CCr	104.48 ± 36.49	97.46 ± 39.01	0.295	0.049
LVEF^a^	1[1,1]	1[1,1]	0.298	0.095
PASP^a^	1[1,1]	1[1,1]	1	0
NYHA	2[2,2.75]	2[1,2]	0.678	0.098
Immobilized	12.5%	15.6%	0.612	0.078
COPD	4.7%	4.7%	1	0
Redo	7.8%	7.8%	1.00	0

AF, atrial fibrillation; BMI, body mass index; CCr, creatinine clearance; COPD, chronic obstructive pulmonary disease; DM, diabetes mellitus; LVEF, left ventricular ejection fraction; PASP, pulmonary artery systolic pressure.

a In the analysis, LVEF was categorized as an ordinal variable using thresholds of >50%, 31%–50%, 21%–30%, and ≤20%; PASP was transformed into an ordinal variable with gradations of Normal, 31–55 mmHg, and >55 mmHg.

### Intergroup comparison

Following propensity score matching (Table 3), patients with TAK experienced a significantly higher incidence of wound complications than matched controls (17.2% vs. 0%, *P* = 0.003), indicating substantially greater wound-related morbidity despite baseline characteristics. Cardiopulmonary bypass times were numerically longer in the TAK group, the difference did not reach statistical significance (143.3 min vs. 130.7 min, *P* = 0.105). Early mortality rates were comparable between groups (3.1% vs. 0%, *P* = 0.480). Importantly, the incidence of postoperative infection was similar between patients with TAK and controls (14.1% vs. 10.9%, *P* = 0.773), despite universal corticosteroid exposure and the potentially higher immune dysregulation in the TAK group. Rates of other perioperative complications were similar.

**Table 3:** Inter-group comparison.

Variables	TAK	NTAK	*P* value
Death in 30 days	3.13%	0	0.48
Dialysis	4.69%	1.56%	0.617
ECMO	1.56%	0	1
IABP	1.56%	0	1
Infection	14.06%	10.94%	0.773
Stroke	1.56%	0	1
Wound problem	17.19%	0	0.003^a^
Aorta clamp (min)	100.3 ± 48.9	92.1 ± 37.7	0.199
CPB (min)	143.3 ± 58.4	130.7 ± 47.6	0.105
Follow-up (year)	4.1 ± 2.6	4.1 ± 2.7	0.938
Follow-up death (HR)	0.667 (0.183–2.422)	1	0.71
Readmission (sHR)	3.487 (1.414–8.559)	1	0.007^a^
NewCVS (sHR)	6.342 (1.325–30.347)	1	0.021^a^
Reoperation (sHR)	1.077 (0.151–7.705)	1	0.941

CPB, cardiopulmonary bypass; CVS, cardiovascular surgery; ECMO, extracorporeal membrane oxygenation; IABP, intra-aortic balloon pump.

a The difference is statistically significant.

**Figure 3:**
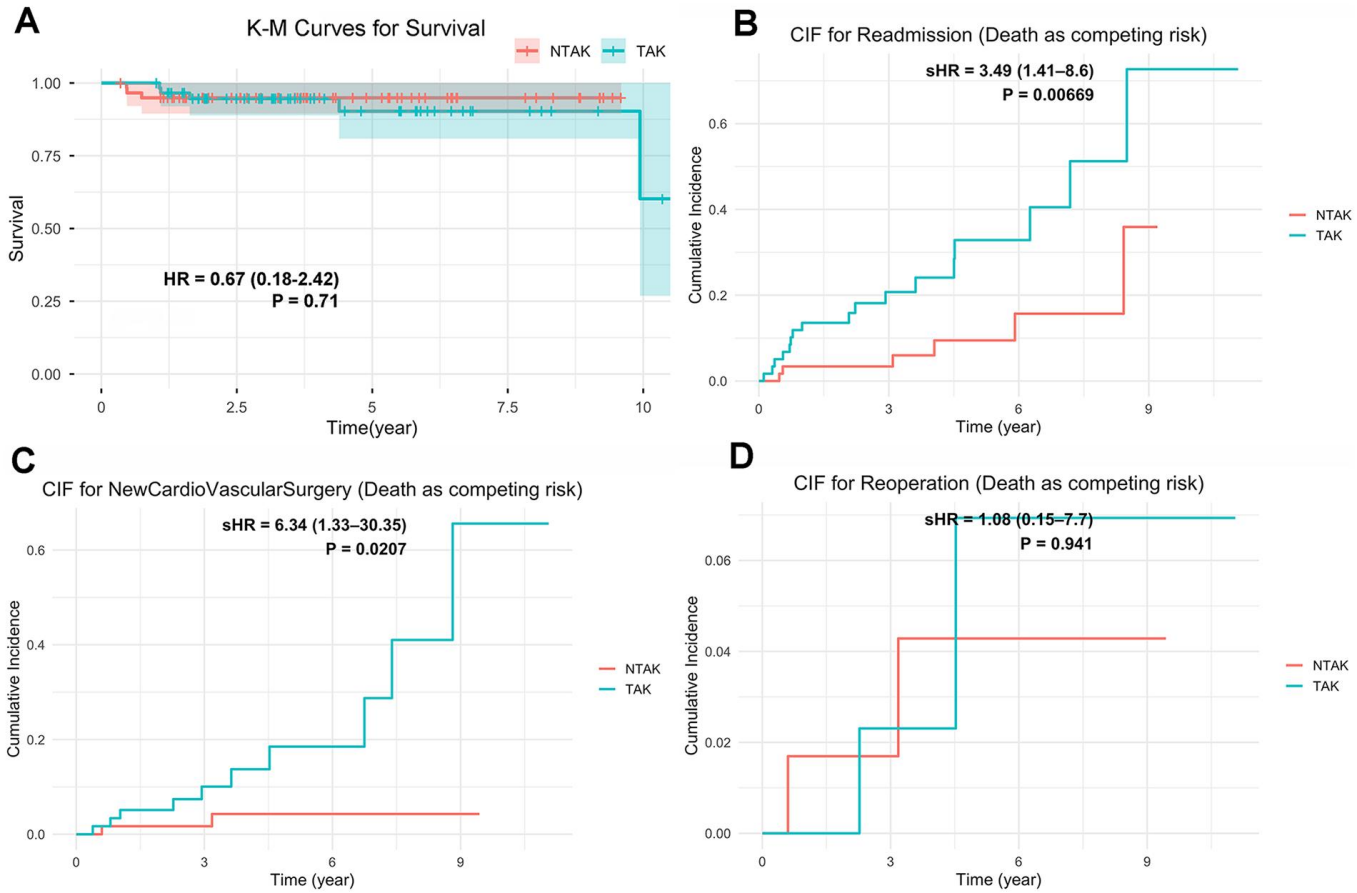
**(A)** Kaplan–Meier curves comparing postoperative survival between the TAK group and NTAK group demonstrated no significant difference between groups (HR, 0.667; 95% CI: 0.183–2.422; log-rank *P* = 0.71). **(B)** CIF curves showed the TAK group had a significantly higher risk of cardiovascular readmission (sHR, 3.487; 95% CI: 1.414–8.559; Gray's test *P* = 0.007). **(C)** CIF curves showed the TAK group had significantly increased risk of requiring new cardiovascular surgery (sHR, 6.342; 95% CI: 1.325–30.347; Gray's test *P* = 0.021). **(D)** CIF curves showed reoperation risk related to the initial surgical failure was not significantly different between the two groups (sHR, 1.077; 95% CI: 0.151–7.705; Gray's test *P* = 0.941).

Critically, survival analysis (Figure 3) demonstrated no significant difference in mortality risk between patients with TAK and matched controls during follow-up (HR, 0.667; 95% CI: 0.183–2.422; *P* = 0.71). However, despite comparable survival, the long-term disease burden diverged significantly. After accounting for mortality as a competing risk, patients with TAK exhibited significantly increased risks of cardiovascular readmission (sHR, 3.487; 95% CI: 1.414–8.559; *P* = 0.007) and of requiring new cardiovascular surgery (sHR, 6.342; 95% CI: 1.325–30.347; *P* = 0.021). In contrast, the risk for reoperation attributable to failure of the initial surgery did not differ significantly between groups (sHR, 1.077; 95% CI: 0.151–7.705; *P* = 0.941).

Notably, unlike TAK-associated aortopathy observed in the study cohort, the matched control cohort exhibited heterogeneous etiology, primarily including bicuspid aortic valve (BAV), secondary hypertension, and familial hypercholesterolemia.

### Prognostic factor analysis for the TAK group

**Figure 4:**
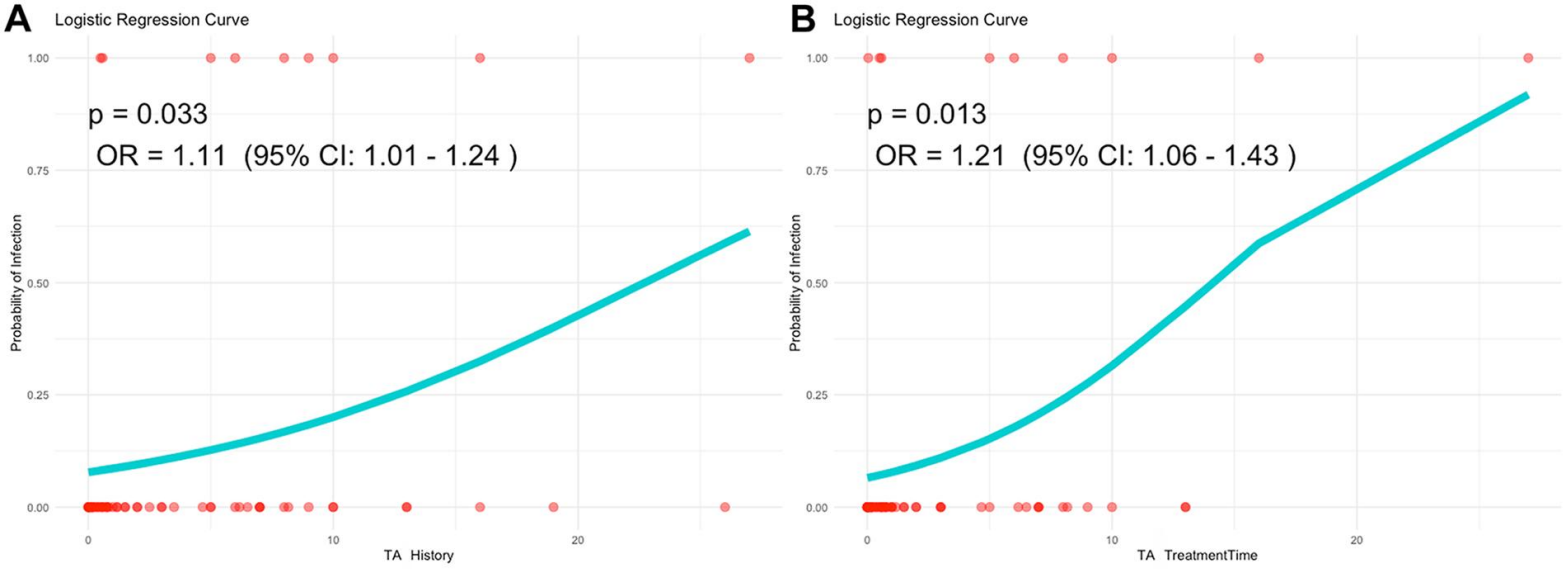
Univariate logistic regression analysis of infection risk factors. **(A)** Association between TA history and infection risk (OR 1.1, 95% CI: 1.01–1.24, *P*=0.033). **(B)** Association between TA treatment duration and infection risk (OR 1.2, 95% CI: 1.06–1.43, *P*=0.013).

Univariate analysis within the TAK cohort identified only two factors associated with an increased risk of postoperative infection (Figure 4): TAK disease duration > 1.75 years (odds ratio [OR] = 1.1, *P* = 0.033) and preoperative TAK treatment duration > 0.75 years (OR = 1.2, *P* = 0.013).

**Figure 5:**
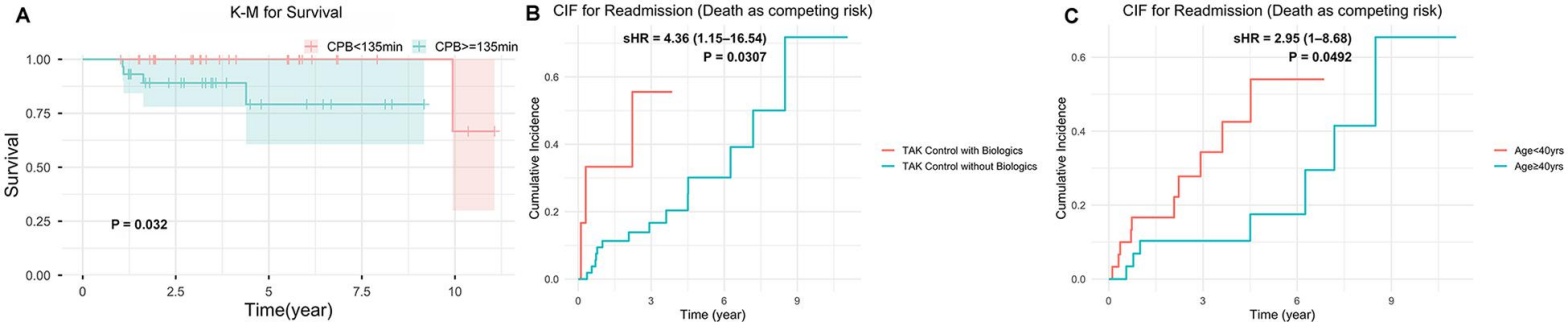
Prognostic factor analyses in the TAK group: **(A)** Kaplan–Meier curves demonstrating significantly lower survival probability in patients with TAK requiring CPB ≥135 min (log-rank *P* = 0.032). **(B)** CIF curves indicating higher readmission risk in patients with TAK receiving bDMARDs preoperatively (Gray's test *P* = 0.031). **(C)** CIF curves showing elevated readmission risk in patients with TAK younger than 40 years (Gray's test *P* = 0.049).

Survival outcomes were analyzed using Kaplan–Meier (Log-rank) and CIF (Fine–Gray) curves. Continuous variables were dichotomized according to mean or median values. Significant findings (Figure 5) demonstrated that cardiopulmonary bypass (CPB) time ≥135 min (*P* = 0.032) was associated with worse postoperative survival. In addition, preoperative bDMARD use (*P* = 0.031) and younger age (<40 years, *P* = 0.049) were associated with significantly increased risks of cardiovascular readmission.

Despite excluding patients with progressively increasing inflammatory activity, at least one abnormal inflammatory biomarker remained present in 39 of 64 patients with TAK (60.9%). Notably, this subgroup demonstrated short- and mid-term outcomes than patients with normal inflammatory markers. Similarly, despite eight patients (12.5%) with histopathological signs of active arteritis in the surgical specimens, no significant differences were observed in their early/mid-term mortality or complication rates compared to TAK patients without pathological activity.

## Discussion

This study included 64 patients with TAK who underwent multidisciplinary preoperative disease activity assessment and control, together with a propensity score-matched NTAK control group. Early mortality did not differ significantly between groups, although patients with TAK experienced a higher incidence of wound complications. Despite perioperative corticosteroid exposure, postoperative infection rates were comparable between TAK and control patients. Mid-term survival was also similar; however, patients with TAK demonstrated higher risks of cardiovascular readmission and new cardiovascular surgeries, whereas the initial operation failure risk remained comparable between groups. Prognostic factor analysis within the TAK group found that longer TAK history and longer TAK treatment duration were associated with an increased risk of postoperative infection. In addition, preoperative use of bDMARDs and younger age were associated with higher risks of cardiovascular readmission. In contrast, no correlation was observed between abnormal inflammatory biomarkers or active pathological findings in surgical specimens and short- to medium-term postoperative outcomes. These findings suggest that assessment of disease activity of TAK should rely on comprehensive clinical evaluation rather than on isolated biomarker values or histopathology findings alone.

It is well established that patients with TAK are at increased risk of aortic diseases requiring cardiac surgery. However, two major clinical questions remain unresolved. The first pertains to the optimal timing of cardiac surgery, particularly regarding preoperative treatment strategies and the assessment of disease activity. The second concerns postoperative prognosis, specifically whether patients with TAK are at increased risk of complications related to surgical failure (e.g., valve detachment, anastomotic leakage, and pseudoaneurysm formation).

In 2005, a study published in *Circulation* reviewed the surgical outcomes of patients with TAK with severe aortic regurgitation (3), finding no association between surgical approaches (valve replacement or composite graft) and the incidence of valve or graft detachment. However, active inflammation was identified in pathological specimens from all patients who subsequently developed detachment, suggesting that persistent local inflammation may contribute to surgical failure. Importantly, this study reflected clinical practice from the 1990s, when multiple antirheumatic drugs for TAK were not yet clinically available, and standardized assessment of disease activity had not been established. Consequently, a substantial proportion of the study population likely underwent aortic surgery during active disease phases, potentially contributing to the observed higher incidence rates of anastomotic detachment.

A subsequent Chinese study evaluating long-term outcomes in patients with TAK with moderate-to-severe aortic regurgitation who underwent either surgical intervention or medical treatment alone (11), demonstrating that surgery not only improved long-term survival but also significantly reduced the risk of adverse events, including non-fatal stroke, myocardial infarction, and congestive heart failure. These findings provide strong evidence for surgical intervention in patients with TAK when appropriate indications are present.

Another Chinese study involving 92 TAK patients with aortic valve disease, including 34 patients who underwent cardiac surgery (2). All patients received preoperative TAK treatment (steroids or DMARDs), and disease activity was assessed using the ITAS-A score. Patients undergoing surgery demonstrated higher ITAS-A scores, reflecting greater disease activity. Beyond variations in preoperative ITAS-A scores, postoperative corticosteroid therapy was partially implemented in the cohort. Consequently, their long-term surgical failure rate was 11.8%. The study also identified a positive correlation between long-term adverse events and the absence of postoperative anti-inflammatory treatment. These findings underscore the importance of adequate preoperative and postoperative control of disease activity.

In 2022, Nam et al. evaluated outcomes in TAK patients who underwent aortic valve replacement (6). Among the 35 patients included in the study, more than half had active TAK preoperatively. The study did not mention the use of DMARDs or postoperative corticosteroid treatment. The NTAK control group was matched using a simple strategy based solely on age. The study found that the TAK group had worse survival rates and required more reoperations during follow-up. Based on our findings, these outcomes may be attributed to uncontrolled disease activity before surgery and the lack of postoperative TAK treatment. In addition, patients with TAK remain susceptible to recurrent cardiovascular events throughout their lives. Importantly, the inability of that study to distinguish revision surgery (for failure of the initial surgery) from *de novo* cardiovascular interventions (for new cardiovascular events) represents a fundamental classification flaw with direct clinical implications.

More recently, Ergi et al. published data on patients with TAK who underwent aortic surgery (5). The study included 31 patients, of whom 83.8% received preoperative steroid treatment, and 70.9% continued steroid therapy postoperatively. All patients were evaluated as having inactive disease preoperatively. Compared with a control group of healthy people matched for age and gender, the study concluded that patients with TAK had significantly worse postoperative survival. This study provides a granular depiction of lesion characteristics observed during aortic surgery and of the ultrastructural features of surgical specimens in patients with TAK. However, its methodologically contentious approach of comparing clinical outcomes with those of healthy controls free from cardiovascular disease substantially undermines the validity of its conclusions—an issue warranting critical scrutiny.

Based on the findings of the aforementioned studies, we introduced a stepwise preoperative medication protocol to achieve disease control before surgery. All patients received standard treatment for TAK and were classified as having inactive disease according to predefined criteria before surgery. Postoperatively, all patients were administered intravenous steroid therapy. For intergroup comparison, an NTAK control group was matched based on identical surgical approaches and the nearest-neighbor propensity scores from a dataset of patients without infectious or autoimmune diseases. Regarding outcomes, we focused on reoperations due to initial surgical failure and new cardiovascular surgeries separately.

Postoperative steroids were implemented to mitigate the risk of adrenal insufficiency from chronic steroid use. We observed no significant difference in postoperative infection rates between the TAK and control groups, despite all patients with TAK receiving perioperative steroid treatment. These results suggest that perioperative intravenous steroid therapy in patients with TAK is safe with regard to infection risk.

Within the TAK cohort, all DMARDs were rigorously withheld preoperatively per institutional protocol until complete wound healing. We therefore attribute the significantly higher incidence of impaired wound healing in patients with TAK primarily to chronic corticosteroid therapy. Consequently, although no excess early postoperative infections were observed, wound complications and related secondary infections require heightened vigilance in TAK cases.

In the prognostic factor analysis, we found that longer TAK history and extended TAK treatment duration were associated with an increased risk of postoperative infections, suggesting that although short-term postoperative steroid use does not increase infection risk in patients with TAK, prolonged disease duration and extended treatment time for TAK still compromise immune status.

We also found that the use of bDMARDs was positively associated with a higher rate of cardiovascular readmissions. The use of bDMARDs typically indicates that disease activity is difficult to control with steroids alone or with csDMARDs. Therefore, it is reasonable to infer that more active TAK may increase the incidence of cardiovascular events and hospital admissions.

### Limitations

The limitations of this study mainly arise from five points: (1) Excessive confounding factors affecting cardiac surgery outcomes coexisted with a substantially limited cohort size. Although propensity score matching was applied to mitigate bias, residual confounding undermined the reliability of the findings. This methodological limitation is particularly salient in prognostic factor analyses within the TAK cohort. (2) The single-center retrospective design. All patients with TAK received preoperative medication, disease activity assessment, surgical interventions, and perioperative management exclusively at one institution. These factors may limit the generalizability of our findings to broader populations. (3) Significant heterogeneity existed in the types of cardiac surgeries performed among the study population, which, combined with the small sample size, further confounded the analysis. (4) Although matching eliminated disparities in surgical procedures between groups, substantial differences persisted in the underlying pathologies requiring aortic surgery during younger periods in TAK versus non-TAK patients, compromising intergroup comparability to some extent. (5) Emerging imaging techniques, such as FAPI–PET/CT, were increasingly employed for preoperative assessment of TAK during the latter phase of the study period. However, as only subgroups of patients underwent this imaging modality, the PET/CT findings were excluded from analysis.

## Conclusions

With multidisciplinary management comprising stepwise pharmacotherapy, standardized activity assessment criteria, and postoperative intravenous steroids, cardiac surgery in carefully selected patients with TAK does not show elevated mortality or morbidity rates. While patients with TAK demonstrate higher rates of cardiovascular-related readmissions and lifelong need for additional cardiovascular interventions, their reoperation risk due to initial surgical failure remains comparable to that of non-TAK patients.

## Data Availability

The raw data supporting the conclusions of this article will be made available by the authors, without undue reservation.
