# Supplementary material for: Assessment of disease activity control and evaluation strategy in patients with Takayasu arteritis undergoing cardiac surgery: a retrospective cohort study
Source: Front Cardiovasc Med. 2026 Jun 3;13:1770394. doi: 10.3389/fcvm.2026.1770394 (PMC13271925; doi:10.3389/fcvm.2026.1770394)
Supplement: Supplementary file 1 [file Datasheet1.docx]

**Supplemental Material**

**Table S1: Codes of different surgeries for TAK paitents**

| **Surgery** | **Code** | **Number** |
| --- | --- | --- |
| AVR | 1 | 23 |
| CABG | 2 | 7 |
| Bentall | 3 | 8 |
| Wheat | 4 | 11 |
| AVR+MVP | 5 | 4 |
| AAR+HemiArc | 6 | 4 |
| Bentall+CABG | 7 | 1 |
| Bentall+TotalArc+ Trunk | 8 | 1 |
| Valsava Sinus Rupture Repair | 9 | 1 |
| AVR+CABG | 10 | 1 |
| Wheat+CABG | 11 | 1 |
| TDAR | 12 | 1 |
| MVP | 13 | 1 |

**Table S2: Definition and data type of the predictors**

| **Predictors** | **Classification** | **Definition** | **Type** |  |
| --- | --- | --- | --- | --- |
| TAK | Main | TAK diagnosis, yes or no | Binary |  |
| TAK history | ‘Dose and time’ issues | Years after the initial diagnosis of TAK | Continuous |  |
| TAK treatment |  | The most aggressive regimen ever used for the treatment of TAK before admission, including corticosteroids, csDMARDs, and boDMARDs | Ordinal |  |
| TAK treatment duration |  | The length of the patient’s last regular treatment of TAK | Continuous |  |
| Peripheral Vascular Stenosis |  | Severe peripheral vascular stenosis evaluated by ultrasound or CTA | Binary |  |
|  |  |  |  |  |
| Biological Markers |  | CRP (ug/ml), ESR (mm/h), TNF-a (nmol/L), and IL-6 (pg/ml) | Continuous |  |
| Pathological Active | Pathological findings of surgical specimens | Granulomatous lesions with transmural inflammation and destruction of muscle-elastic lamina within the tunica media. | Binary |  |
